# On the Position of Fractured Limbs

**Published:** 1820-08

**Authors:** 


FOR THE LONDON MEDICAL AND PHYSICAL JOURNAL.

### *On the Position of Fractured Limbs.* By Dr. Kinglake.

THE controversial and practical differences of opinion and conduct that have long subsisted respecting the treatment of fractured limbs, particularly of the lower extremities, may yet be regarded as *sub judice.* Such a decision as would prove an unerring guide in determining the management of these cases, is still wanting; or, at least, it has not been so unequivocally given as the importance of the subject demands. Whether the broken limb should be made to assume a curved or straight direction, continues to be the question at issue. Much has been already said on this subject; and the advocates of the different modes of treatment have respectively asserted, and endeavoured to maintain, the grounds on which is rested the difference of opinion.

The earliest periods of practical surgery must have been those in which theoretical consideration could have had but little influence; the suggestions of what appeared to be agreeable to common sense, and to be obviously necessary from the state of circumstances, were then adopted, as the most natural and certain rule of conduct. To have insisted at that time, that laying a broken bone in a curved position would be favourable both to the osseous union and to the eventual straightness of the limb, would have been thought unaccountably strange, and altogether untenable. The familiarities, the prejudices, and the satisfactory results, of long-established practice, would have caused the proposition to be regarded as rather chimerical than rational, and it would probably have been disposed of *sub silentio*. It is hardly to be conceived that it would have been entertained as a subject admitting of any serious discussion, but that it would have been at once held to be absolutely inadmissible. The refinements of physiological consideration, the estimates made of motive power, and more especially of the voluntary and involuntary action of the muscular structure, furnished an ample field for speculating on causes and effects that might be supposed to be influential in relieving and curing fractured limbs. In this course of theoretical enquiry it occurred, that a relaxed state of the muscles which are directly concerned in moving the limb that may have been broken, would lessen the excitement that must inseparably accompany an extended condition of such muscles. This is plausible in theory: it would even appear to be authorized by physiological estimations of the inevitable effects of muscular action on the fractured ends of bones. If this theory, however, be examined by facts, (the only test by which the validity of all speculations should be tried,) it will be found that the speciousness of hypothetical expectation is too much at variance with practical results, to be entitled to confident admission.

In regarding the curved position of a fractured limb as most favourable to its speedy and perfect restoration, it is imagined, that a relaxed state of the muscles connected with its motive powers is a condition of ease and freedom from all undue excitement. Were this the case, it would go far in reconciling the objection to the practice; but the reverse is most commonly the fact. The range of involuntary action given to the muscles connected with a fractured bone when in a relaxed state, is such as operates most injuriously on the stationary position to which it would be desirable constantly to confine the broken ends of the bone. An action, which may be called spasmodic, or even convulsive, is very apt to be induced in the irritable circumstances almost invariably attending the mischievous violence inflicted, as well on the broken bone as on the surrounding muscular parts. The pain necessarily prevailing in the inflamed portions of the broken bone is sympathetically communicated to the adjoining muscular structure, by which it is thrown into irregular actions, that cannot fail to have a deranging effect on the mechanical position or co-aptation of the ends of the broken bone. This will have the effect of disturbing any union that may have commenced; and, by its disjoining influence, to aggravate the attending inflammation, to protract the period of hurtful irritation, and even, eventually, to occasion a mis-shapen, distorted, and an imperfect, union of the broken bone. If, instead of this unconfined sphere of action afforded by the relaxed state of muscles in the curved position of a broken bone, it were abridged and limited by the straight posture, in which the broken ends of the bone might be supported and preserved in lineal contact by the constraint of suitable bandaging, it is reasonable to expect, that much distressing and hurtful irritation would be effectually obviated. Experience demonstrates the truth of this observation; and facts prove, beyond contradiction, how much more readily and salutarily the usually-occurring difficulties attending fractures are, under the control of a straight position, confining and preserving the affected parts in a comparative state of mechanical ease and quietude, than when the curved posture has loosened and left at greater liberty the broken ends of the bone, as well as the adjacent muscles.

The scheme of using splints long enough to comprehend the joints above and below the fractured bone, is fallacious. Whatever fixation or steadiness such splints may impose on the joints, they cannot sufficiently affect the intermediate parts, in which the fracture and surrounding muscles are comprised. These parts are not subjected to the check and confinement that are requisite to prevent the hurtful derangement to which they are liable, from the involuntary and disturbing muscular action that must necessarily be going on in such circumstances of unrestricted freedom. If steadiness of position be not preserved in a broken limb, how can it be reasonably expected that the fractured ends of the bone can perfectly unite? and, if the process be repeatedly disturbed and delayed, the result will probably be an imperfect restoration of the limb, exhibited in either a deviation from the straight line at the fractured part, in a protuberant accumulation of osseous matter about or around the broken part, or in a shortening of its natural length, by a splicing or overlapping of the fractured ends of the bone. In either of these cases, an incomplete cure must be the result. The limb is either curved and disfigured at the fractured part, or it is so shortened, as greatly to injure its natural capability of motion. Another, and a very serious objection, that might be urged against the practice of placing a fractured limb in a bent position, is the liability that is incurred of having the broken ends of the bone so often disturbed as, at least, to prevent an early ossific union at the fractured part. When the exudation of osseous matter in the incipient stage of that process is checked and diverted from its regular course, much evil is incurred. The violence attending such an interruption induces additional inflammation, which may terminate in either gangrene, suppuration, or carious decomposition of the bony structure, with ulcerative destruction of the superincumbent soft parts. One of the mischiefs here stated as incidental to a frequent displacement of the ends of a broken bone, is sometimes injudiciously and irreparably occasioned by the absurd practice of aiming at salutarily stimulating the surfaces of the broken ends, by moving them at intervals on each other, with a view to the exciting influence of attrition.

It has occurred to me to see this practice injuriously pursued, at longer or shorter intervals, for weeks, nay, in one case for months, which actually issued, and that too almost inevitably, in a sort of cartilaginous flexible union of the broken bone. What can be so preposterous as to superadd to natural processes for the restoration of parts, the crude suggestion of an hypothesis unwarranted by any applicable analogies or reasonable calculations of eventual benefit? The vital and restorative agencies of nature can only obtain in certain relative circumstances; and, if these should be materially infringed, the designed effect cannot take place, but a perverted and morbid one must ensue. A mode of treatment, therefore, that would be at all likely to involve such consequences, must be defective in some of the considerations on which its uniform power is supposed to rest. The grand defect in the scheme of assisting recovery in fractures by the bent position or relaxed state of the muscles connected with the broken limb, is, that it has an irresistible tendency rather to prevent than to secure the motionless rest that is so essential to a perfect cure in such cases. The object of the proposition is to effect that which cannot be accomplished in the way stated; it is therefore a serious practical injury to supersede a mode of treatment unequivocally successful, and, generally speaking, equal to all the curative advantages that could be reasonably desired by an innovation founded more on ingenious speculation, than on demonstrative proof of superior benefit.

The error into which it would seem the advocates for the bent position first fell, and by which they continue to be misled, is the strange supposition, that relaxed muscles would remain in such a quiescent or inactive state, as not to divert from the natural line the ends of a broken bone with which they were connected. This is reasoning rather on the properties of inanimate than of living matter: the inertion of the former would insure sufficient stillness in any mechanical arrangement that might be proposed; but, in the latter, the circumstances are much too sentient, and the disturbance too great, to admit of the possibility of preventing those irregular or spasmodic actions which at once move both the upper and lower joints, as well as every other part of the broken limb. Not only is motion inevitably induced, but, even what may be considered as a convulsive shudder, often pervades the limb, which would almost unavoidably displace the fractured ends at the early stages of ossific union, or, in certain cases, wholly prevent any effectual junction from taking place. The admirable provision made by nature for the restoration of parts in the animal economy from the destructive effects of disease, renders it difficult, even under gross mismanagement, to prevent its being operative to a greater or less extent; so that the result is at least an approach to a cure, where the salutary design may have been frustrated by adventitious impediments. To experience, as the surest test of what is well founded, must an appeal be made in all controverted and doubtful questions. To this authority may be safely left the preference due to the straight over the bent position, in the treatment of fractured limbs.

The straight position is far from being one of ease and comfort; it is inseparably connected with a certain extent of pain and suffering: but the question here is, whether that degree of pain and suffering is not much less severe in its immediate experience, and fraught with less mischievous consequences, than may be expected to arise from the bent posture? That the affirmative of this question is true, cannot be justly denied: it is therefore warrantable to prefer and adopt the greater benefit presented by the two different modes of affording relief. No dispute into which the patrons and promoters of the bent position may be inclined to enter, can alter the incontrovertible facts of the case. Much ingenuity, great learning, and an inflexible adherence to an accustomed opinion, may be shown; but still the real merits of the subject will remain unshaken, and be entitled to all the consideration that is due to demonstrable truth,—to a practical advantage, that realises every reasonable hope and calculation that could be entertained on the occasion.

The late Mr. Pott, the great patron of the bent position in fractured limbs, and numerous other advocates of the practice, would seem to have strangely miscalculated the comparative liabilities which the broken ends of bones were under to being moved in the straight and bent postures, in supposing that they were greater in the former than the latter. Whatever fixation may be given to both the upper and lower joints of the broken limb, it is almost impossible to preserve a motionless state of the fractured parts, without imposing immediately on them the stricture of suitable bandaging. Indeed, the mechanical security that is imagined to be afforded by confining the joints and relaxing the muscles, is fallacious and unattainable, independently of the movements that must often be occasioned by the involuntary action of muscles, suffering either from contusion or from sympathetic excitement. The proposal that has been made, of placing the fractured limb at first in the bent position, with a view to relax the muscles, and thereby either to expedite the removal, or to prevent the occurrence, of inflammatory tension, is likewise deceptious, and is not justified by practical experience. If the broken ends of the bone be not duly replaced, tension and pain will necessarily ensue ; and, if they have already occurred, they will be exasperated, by suffering the irritating cause that produced them to continue. Immediate reduction of the fractured bone, whether simple or compound, should be effected, provided the injury sustained by the surrounding parts, and by the bone itself, be not to an extent that would preclude all reasonable prospect of saving the limb ; in which case prompt amputation is the only alternative ; and, if not seasonably resorted to, life itself would be imminently and speedily endangered. When reduced, the fractured limb should be laid in the position that appears to be best adapted to keeping it most free from hurtful motion.

The straight posture, with all due deference to the ingenious speculations that have been indulged in favour of a bent and relaxed state of the limb, is certainly authorised by a vast extent of most gratifying success. The sanction of ages had deservedly conferred on the straight position an unimpeachable reputation for salutary efficacy. It did accomplish, and still effects, all that is desirable in these cases. What then are the deficiencies that need be supplied ; and how are they furnished by hypothetical refinements, on which the claims of the bent position would seek to be preferred ? In compound fractures, attended with much contusion, and even threatening laceration of the soft parts, the most effectual and speedy cure is obtained by reducing the fracture as soon as possible, and by binding up the soft parts with sufficient strictness to keep the broken ends in contact. The exclusion of atmospheric air is of the utmost importance in promoting and facilitating the process of healing and regenerating injured and mutilated parts. It induces, as it were, union by the first intention, and obviates the harassing and eventful procedure of sloughing, abscesses, and exfoliations.

The prevailing custom of frequently inspecting compound fractures, and more particularly the inconsiderate and unfeeling practice of trying at intervals if the broken ends of the bone move on each other, are apt to produce serious injury. The process of recovery instituted and pursued by the natural capabilities of the affected parts, should not be thwarted by factitious endeavours to direct and regulate these inherent operations. Mechanical obstacles are always fit objects for the adjusting interference of art: if they be not seasonably removed, irreparable mischief may be incurred by the delay; and, during the continuance of such impediments, no cure can be effected. In the management of fractured bones, therefore, the chief object is to place the injured parts, as early as circumstances will admit, as much as possible in their natural situation, and then to leave the progress of restoration to the healthy state wholly to the resources and agencies of nature. It is not precisely known in what the regenerative processes consist, or how they act in their salutary work of uniting and reproducing lost substances; but it is most clear, that natural temperature, the avoidance of unnecessary handling and unavailing topical applications, sustaining due pressure by means of appropriate bandaging, and, above all, preserving with the utmost care as unmoved a stillness of the fractured limb as possible, are absolutely necessary in the treatment of broken bones. These advantages are best obtained by so arranging the disturbed parts, in the first instance, as to render any considerable interference at subsequent periods unnecessary. All that is required to be done, after the fractured limb has been mechanically secured, must be effected by the restorative power of the affected parts themselves: it is therefore the legitimate province of art, and the duty of scientific surgery, to co-operate in the intention of nature less by positive than by negative assistance, in withholding whatever may be hurtful, and merely supplying the aid that is obviously and indispensably necessary.

*Taunton; May 14th*, 1820.

